# Prophylactic and Therapeutic HPV Vaccines: Current Scenario and Perspectives

**DOI:** 10.3389/fcimb.2022.909223

**Published:** 2022-07-04

**Authors:** Yicheng Mo, Jiabing Ma, Hongtao Zhang, Junjie Shen, Jun Chen, Juan Hong, Yanmin Xu, Cheng Qian

**Affiliations:** ^1^ College of Life Sciences and Medicine, Zhejiang Sci-Tech University, Hangzhou, China; ^2^ IND Center, Chongqing Institute of Precision Medicine and Biotechnology Co., Ltd., Chongqing, China; ^3^ IND Center, Chongqing Precision Biotech Co., Ltd., Chongqing, China; ^4^ Center for Precision Medicine of Cancer, Chongqing Key Laboratory of Translational Research for Cancer Metastasis and Individualized Treatment, Chongqing University Cancer Hospital, Chongqing, China

**Keywords:** HPV, cervical cancer, pathogenic mechanism, prophylactic vaccines, therapeutic vaccines

## Abstract

Persistent human papillomavirus (HPV) infection is recognized as the main cause of cervical cancer and other malignant cancers. Although early detection and treatment can be achieved by effective HPV screening methods and surgical procedures, the disease load has not been adequately mitigated yet, especially in the underdeveloped areas. Vaccine, being regarded as a more effective solution, is expected to prevent virus infection and the consequent diseases in the phases of both prevention and treatment. Currently, there are three licensed prophylactic vaccines for L1-VLPs, namely bivalent, quadrivalent and nonavalent vaccine. About 90% of HPV infections have been effectively prevented with the implementation of vaccines worldwide. However, no significant therapeutic effect has been observed on the already existed infections and lesions. Therapeutic vaccine designed for oncoprotein E6/E7 activates cellular immunity rather than focuses on neutralizing antibodies, which is considered as an ideal immune method to eliminate infection. In this review, we elaborate on the classification, mechanism, and clinical effects of HPV vaccines for disease prevention and treatment, in order to make improvements to the current situation of HPV vaccines by provoking new ideas.

## 1 Introduction

Cancer is a disease with a high mortality rate, and many people develop cancer every year, including cervical cancer and other conditions caused by HPV infection. For HPV prevention, periodic physical examination is important, while vaccination is the most economical and effective way. The currently available HPV vaccines are designed using a combination of multiple subtypes of L1-VLPs, and the L1 spontaneously formed VLPs are highly immunogenic and produce high titers of neutralizing antibodies to prevent HPV infection (Stanley et al., 2021). Since 2006, the bivalent, quadrivalent and nonavalent vaccines have had relatively good preventive effects. However, there are corresponding problems, one is the high cost of L1 VLP vaccine with *Saccharomyces cerevisiae* as the expression system, and the other is that L1 VLP has no cross-protection and can only prevent multi-subtype HPV infection by increasing the VLP type, which is the reason for the high price of the vaccine. However, there is a novel target antigen L2, which is concerned with using the broad-spectrum protection of L2 to provide prevention against multiple subtypes (Kumar et al., 2015; Huber et al., 2021). However, its immunogenicity is poor and the induced neutralizing antibody titers are much lower than those of L1 VLP, so improving its immunogenicity is the primary problem to be solved in the development of L2 vaccine (Tyler et al., 2014). This paper provides an overview of the current status of HPV prophylactic vaccines and an outlook on the future direction of HPV prophylactic vaccine improvement.

For the treatment of cervical cancer and other HPV-associated cancers, surgery remains the dominant treatment. Therapeutic vaccines can be developed both as mainstream treatment and as an adjunct to surgery to improve treatment outcomes and prevent recurrence. There are different types of HPV therapeutic vaccines that are expected to elicit better immune effects including Live vector-based vaccines, protein vaccines, nucleic acid vaccines and Whole cell vaccines. The starting point of most of these vaccines is focused on the E6 and E7 antigenic targets and are designed to activate systemic cellular immunity and kill HPV-infected cells through the CTL response (Chabeda et al., 2018). However, many vaccines that achieved satisfactory preclinically results do not work well in the clinic, and there is still a need to optimize current vaccine regimens and to develop new vaccines. This paper reviews the current classification of HPV therapeutic vaccines and the preclinical and clinical effectiveness of each vaccine, and provides an outlook on future vaccine development, such as evaluating the impact of multiple tumor settings on vaccine efficacy and the availability of new antigenic targets that could enhance vaccine immunogenicity.

## 2 HPV and Cervical Cancer

**Figure 1:**
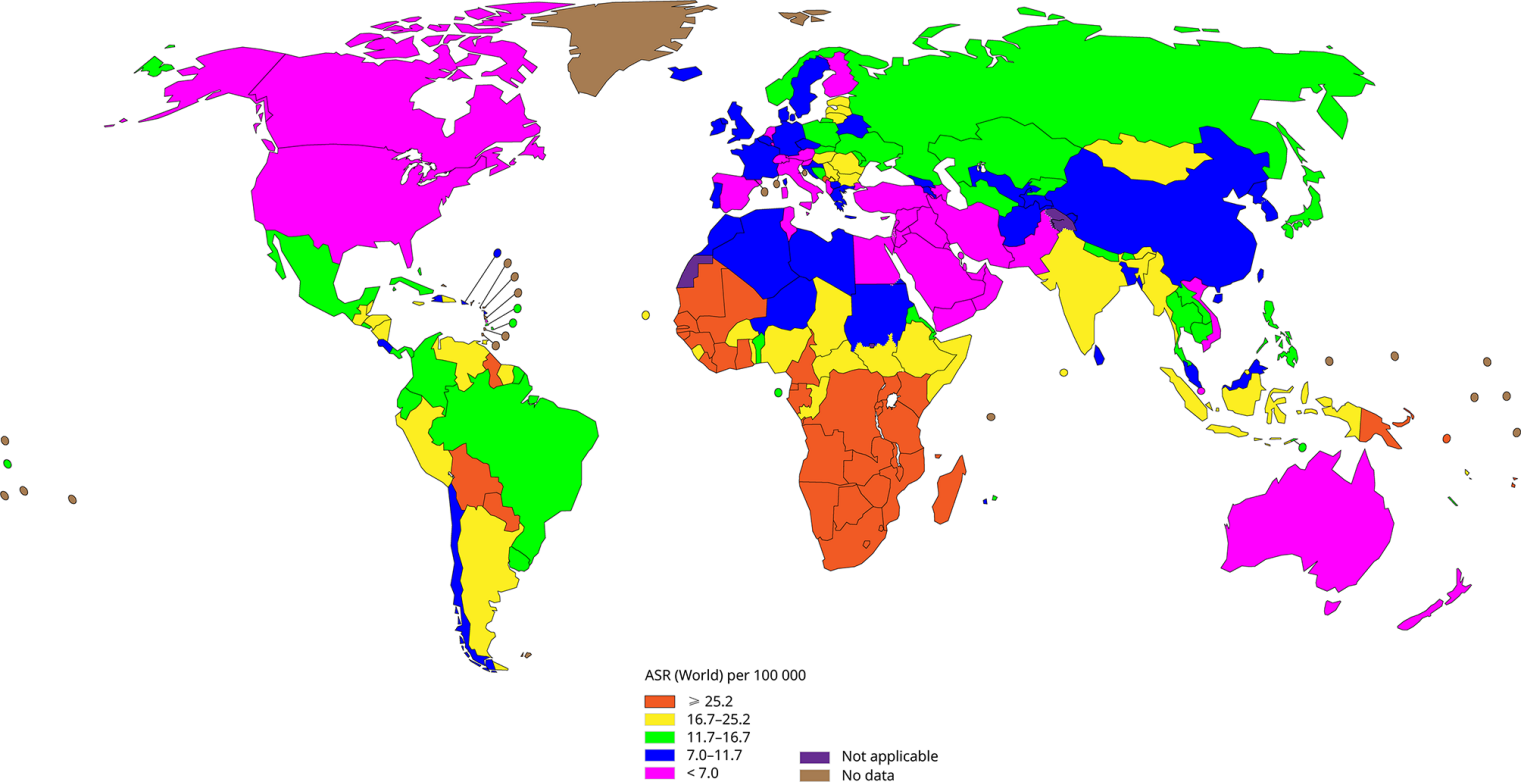
The incidence of cervical cancer in all regions of the world (Data come from WHO, 2020), the incidence of cervical cancer is lower in developed regions such as North America and Europe, and higher in backward regions such as Africa.

Human papillomavirus (HPV) infection induces almost all cervical cancers and it is related to about 90% of anal cancer, 50% of penile cancer, 70% of vagina cancer and 20-60% of oropharyngeal cancer, among which cervical cancer is one of the most common cancers that threaten women’s health, besides it is the fourth most common cancer among women worldwide (Forman et al., 2012). **Figure 1** shows the incidence of cervical cancer in various regions of the world according to WHO statistics in 2020. Cervical cancer accounts for 3.1% of all cancers, with about 604,127 new cases and 341,831 deaths annually (Hu and Ma, 2018). It is worth noting that in developed countries, with the advancement of screening methods and the popularization of vaccines, the incidence and mortality of cervical cancer have gradually decreased (Siegel et al., 2016; Pinatti et al., 2018). While for poor and populous developing countries, laggard screening methods and high vaccination costs have made cervical cancer the second most common cancer in women.

Cervical cancer originates from normal cervical epithelium, upgrading from mild cervical intraepithelial neoplasia (CIN 1) to more serious invasive neoplasia (CIN 2 or CIN 3) and eventually progressing into invasive cancer (Burd, 2003). At present, it is generally believed that the occurrence of cervical cancer is related to factors such as HPV persistent infection, and somatic mutation of host genome. Persistent infection of high-risk HPV (HPV16/18) is the most essential factor in the development of cervical cancer. The virus is transmitted primarily through sexual intercourse and prevails in sexually active women and men with genital infections, but these common infections do not induce the development of serious cancers. Data shows that 60% of human papillomavirus infection in one year will be naturally cleared, 90% of HPV infections tend to be cleared within two years, while only a very small number of cases progress into precancerous lesions and cancer (Moscicki et al., 2004). Natural infection induces low levels of specific neutralizing antibodies against the viral capsid protein (L1). These antibodies are able to prevent some of the infection, especially in those with the highest antibody titers. But when the infection continuously exists and immune system loses control, dysregulation of viral gene expression, cell proliferation and the accumulation of genetic damage can lead to precancerous lesions and cancer within a few years.

The occurrence and progression of cervical cancer can be detected by hrHPV (high-risk types of HPV) testing, Pap smears and colposcopy alone or in combination to measure the extent of high-risk HPV integration and precancerous lesions in the cervical epithelium (Pierce Campbell et al., 2012; Schiffman and Solomon, 2013). The most effective way to prevent cervical cancer and HPV-related infections is vaccination, and several available HPV vaccines have achieved good results (Spinner et al., 2019). For patients with further infection, surgical treatment is the mainstay. In the early stage of precancerous lesions, conization and loop electrosurgical excision (LEEP) are effective methods (Santesso et al., 2016). After the development of cervical cancer, surgical resection combined with chemotherapy (such as cisplatin-based chemotherapy combined with bevacizumab regimen) shows priority (Tewari et al., 2014; Fader, 2018). However, the risk of surgical treatment remains. For survival after surgery for early-stage cervical cancer, a US study analyzed a 4-year mortality rate of 9.1% in women who underwent minimally invasive surgery (Melamed et al., 2018). For advanced Cervical cancer stage II, III and IV cancers, postoperative follow-up in the UK and Germany reported poor five-year survival rates of 54, 75, 58-63 and 38, 58, 32-35%, respectively. Our ultimate goal is therefore to reduce this risk by developing highly effective therapeutic vaccines.

## 3 Genome and Pathogenesis of HPV

### 3.1 HPV Genotypes and Structures

HPV belongs to the family Papillomaviridae and is a small circular DNA virus with about 8,000 base pairs. At present, more than 200 types of HPV have been found, of which about 40 types are associated with genital infections and classified as high-risk and low-risk according to their carcinogenic potential. 15 types of HPV are categorized as high-risk types (16, 18, 31, 33, 35, 39, 45, 51, 52, 56, 58, 59, 68, 73 and 82), which are related to tissue lesions and aggressive cancers (cervical cancer, head and neck cancer, respiratory cancer); 12 types of HPV are known to be low-risk types (6, 11, 40, 42, 43, 44, 54, 61, 70, 72, 81 and CP 6108), which are mainly related to genital warts and benign cervical lesions (Muñoz et al., 2003). Among them, HPV16 and HPV18 are two most common types, they are associated with about 70 percent of cervical cancers and 80-90% of other HPV related tumors (McCormack, 2014).

**Figure 2:**
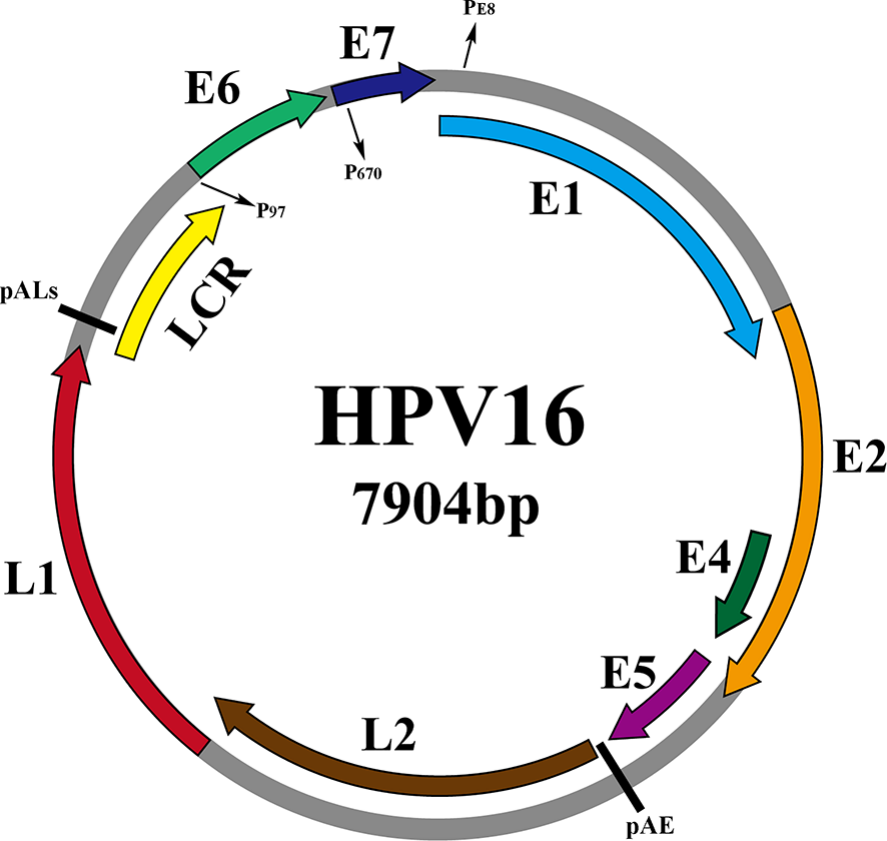
Schematic representation of the dsHPV16 genome (gray circles, other HPV subtypes are similar to it), with the ORFs of the virus indicated by colored arcs above the genome. The promoters are indicated by P (P97, P670, PE8) and the pAE and pALs (polyadenylation sites) stages are indicated by short straight lines.

As shown in **Figure 2**, taking the genome of HPV16 as an example, the HPV genome can be divided into 3 functional regions. Early (E) regions that encode early proteins (E1, E2, E4, E5, E6, E7), plays key roles in HPV genome replication, gene expression and immune evasion during the virus life cycle. The late (L) region that encodes the late capsid protein (L1, L2), is involved in viral infection, delivery and packaging (Moody and Laimins, 2010). And the non-coding region, namely the long control region (LCR), is recognized as the upstream regulatory region (URR), accounts for one-eighth of the entire HPV genome and contains the viral origin of replication (ORI) and early promoter regulatory elements, but does not encode any protein (Albert and Laimins, 2020). **Table 1** shows the specific functions of the proteins

**Table 1:** Early and late protein functions of HPV.

Proteins	Functions
E1	Regulate viral DNA replication
E2	Regulatory factors of viral transcription
E4	Promote virus maturation and release
E5	Regulate growth factor signaling pathway
E6	Promotes the degradation of P53 and increases resistance to apoptosis
E7	Promotes retinoblastoma protein (pRb) degradation, affects the cell cycle and stimulates cell proliferation
L1	Major capsid protein is important for virus assembly and stability
L2	Secondary capsid protein is important for virus infection

### 3.2 Life Cycle and Pathogenic Mechanism of HPV

**Figure 3:**
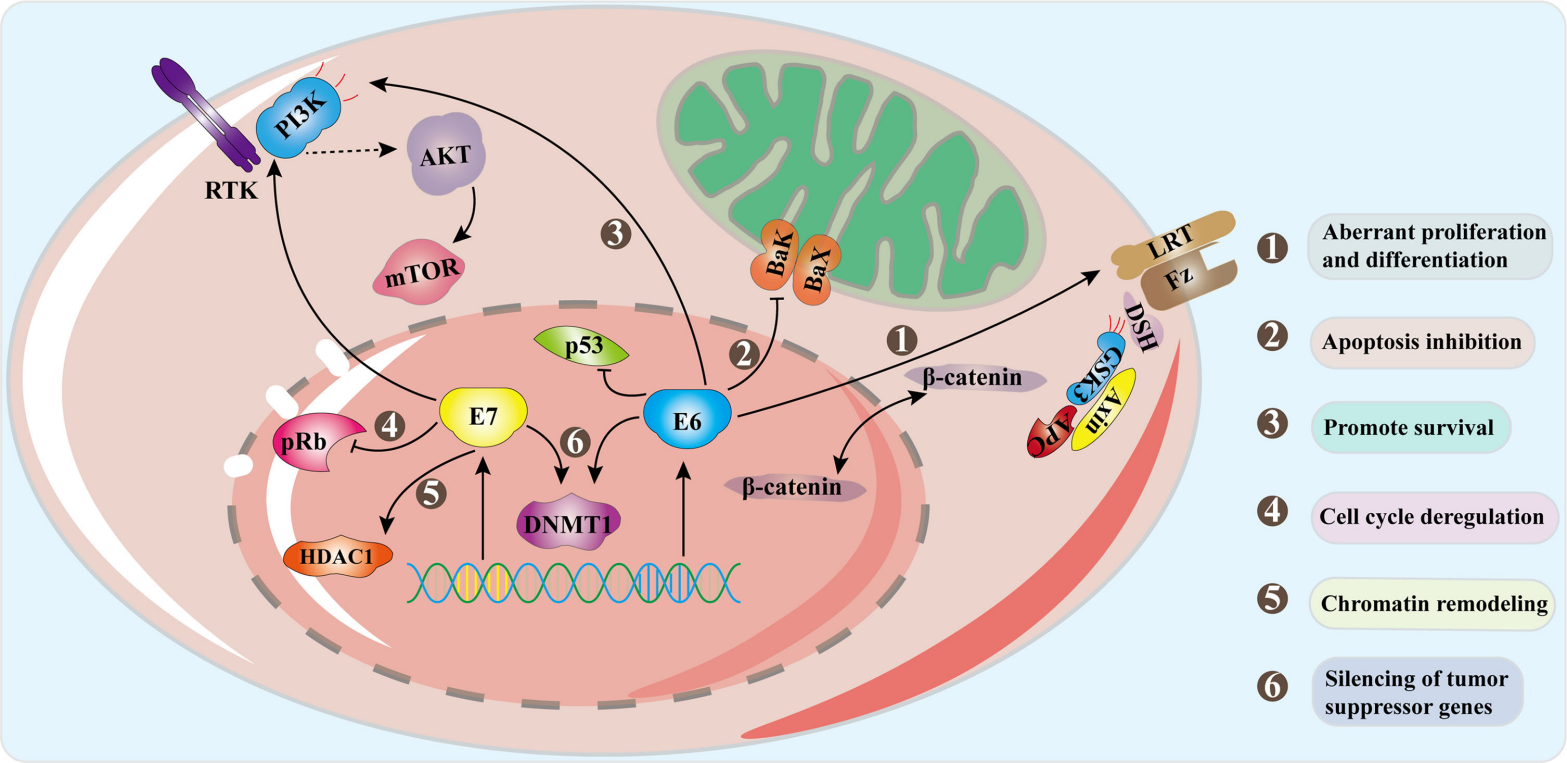
Signal pathways in progression to cervical cancer. Oncogenes E6 and E7 are overexpressed as the HPV genome is integrated into the host genome. E6 combines with E6AP to degrade p53, and E6 activates the Wnt/β-catenin pathway which increases β-catenin and promotes cell proliferation. E7 acts on the Rb family and disrupts their connection with E2F transcription factors. The interaction of E7 with HDACs leads to chromosome remodeling and genome instability. E6 acts on PDZ protein to inactivate PTEN, resulting in an increase in pAkt and enhancing cell proliferation. E7 enhances the ability of Akt to bind and inactivate Rb, which proves that both E6 and E7 can activate the PI3K/Akt pathway. The interaction between the two oncoproteins and DNMTs leads to abnormal methylation and thus silences tumor suppressor genes. The combined effect of these mechanisms causes the occurrence of tumors.

The HPV life cycle is closely related to epithelial differentiation of keratinocytes of the host, and the production phase of the virus life cycle is limited to terminally differentiated epithelial cells (McBride, 2017). First of all, HPV goes through micro scratch into the epithelium and then infect actively dividing basal, or stem, epithelial cells. It then binds to heparan sulfate proteoglycan (HSPG) receptors located on the cell surface of the basement membrane or basal lamina *via* the L1 capsid protein (the epithelial cell receptor of HPV6 is α6-integrin). When HPV binds to HSPG, the viral shell undergoes a cyclophilin B-mediated conformational change, which exposes the N-terminus end of the L2 component to the viral surface. The N-terminus is then cleaved by furin and/or PC5/6 and this allows binding to a secondary receptor on the plasma membrane of the target cell (Egawa, 2003; Pyeon et al., 2009). HPV then enters the cell through an endocytic mechanism that has most similarities with micropinocytosis (DiGiuseppe et al., 2017). Approximately 24 hours after attaching to cells with the assistance of L2 capsid proteins, HPV reaches the nucleus. In the host cell nucleus, the early stage of the virus replication cycle begins with the expression of E1 and E2, and the early expression of the viral transcription factor E2 can correctly regulate the early viral promoters (P97 of HPV 16 and P105 of HPV 18, located upstream of E6 ORF) to ensure the expression of E6 and E7 regulatory proteins, so as to ensure the continuous survival of HPV-infected cells (Graham, 2010; Graham, 2017). E2 combines with E1 and then binds as a dimer of hexamers to the viral origin of replication and recruits the cellular DNA replication machinery. But at this time, the number of copies initially replicated in the host nucleus is very small, with approximately 50-100 copies per nucleus (McBride, 2017). As the infected cells differentiate and enter the later stage of the viral replication cycle, the expressions of early proteins E4 and E5 and the late proteins L1 and L2 are initiated. E4 and E5 also contribute to viral replication, along with high levels of E1 and E2, thus promoting the expansion of the viral genome to thousands of copies per cell. E4 protein may play an important role in reorganizing cytokeratin filaments at the late stage of the virus replication cycle, which makes cells fragile and facilitates the release of offspring virus particles (Graham, 2017). L2 is synthesized before L1 and is transported to the nucleus, while the L1 protein self-assembles into pentamer shells in the cytoplasm (Becker et al., 2003). These structures are transported to the nucleus, where L1 interacts with L2 to assemble into virus particles in the center of the shell, and the new virus particles are subsequently released from the cell with the assistance of E4 protein (Buck et al., 2005). In the whole process, in order to keep the cell replication mechanism active, the expression of oncoproteins E6 and E7 interferes with the cell cycle of the host. E7 activates the cell cycle of infected differentiated cells by binding and degrading pRb from a transcriptional repressor complex containing the E2F transcription factor. E2F becomes free to activate the cell cyclin-dependent kinase 2 (Cdk2)/Cyclin A and Cdk2/Cyclin E complex to eliminate cell cycle arrest, thereby stimulating proliferation (Dyson et al., 1989; Helt and Galloway, 2001). E6 increases resistance to apoptosis and allows viral DNA replication by degrading p53 through E6AP (Arroyo et al., 1993; McIntyre et al., 1996; Gupta and Mania-Pramanik, 2019). Although they are designated as oncoproteins, their expression is essential for the normal replication of the HPV life cycle. Persistent HPV infection will produce precancerous lesions called squamous intra-epithelial lesions (SIL classified as LSIL/HSIL) or cervical intra-epithelial lesions (CIN1, CIN2 and CIN3) (Moscicki et al., 2012). **Figure 3** demonstrates some of the signaling pathways in the development of cervical cancer including Wnt/β-catenin, PI3K/Akt (Bossler et al., 2019; Wang et al., 2020).

## 4 Research Progress of HPV Prophylactic Vaccines

Vaccination is the most effective measure to prevent HPV infection from related diseases. The heterologous antigens carried by the vaccine enter the body, are captured by the APC and introduced into T lymphocytes, and assist B lymphocytes in producing antibodies, which is the main basis for the vaccine prevention effect (de Jong et al., 2004). Antibodies can penetrate the blood vessel wall to reach the infection site, and combine with the virus to weaken ability to infect (Nardelli-Haefliger et al., 2003). However, the immune response produced by human natural infection with HPV is weak and the antibody titer is very low.

Currently, licensed HPV vaccines are developed on the basis of the virus-like particles (VLPs) of the major papillomavirus capsid protein L1, which are empty viral shells made up of one or more types of polymeric shells or capsid proteins (Yadav et al., 2019). VLPs do not contain a viral genome and are therefore not infectious or carcinogenic. Besides they can induce a strong humoral immune response with high and long-lasting neutralizing antibodies (Buck et al., 2013; Cheng et al., 2020).

### 4.1 Current Status of Commercially Available HPV Prophylactic Vaccines

**Figure 4:**
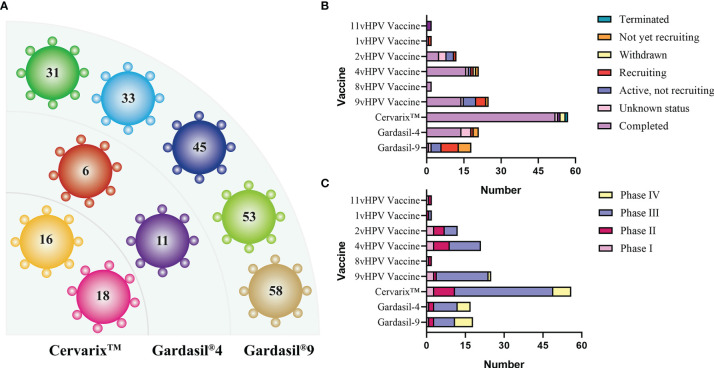
**(A)** HPV genotypes contained in the three prophylactic vaccines. **(B, C)** Status and stage of prophylactic vaccines in clinical trials (data from ClinicalTrials.gov). In addition to the three common vaccines, the current status of other vaccines ranging from 1v to 11v is also sorted out, with the majority of clinical trials completed and many of the incomplete trials being geographic inter-rater effect analyses.

There are three HPV prophylactic vaccines currently available. They are respectively Gardasil^®^4, a quadrivalent vaccine available in 2006, Cervarix™, a bivalent vaccine available in 2007, and Gardasil^®^9, a nonavalent vaccine available in 2014 (Hanna and Bachmann, 2006; Crosbie and Kitchener, 2007; Printz, 2015). All three HPV vaccines were developed based on L1 VLP. Data from clinical trials showed that these three vaccines all achieved good preventive effects on people infected by HPV from different regions, of different races, and in different age groups. Moreover, the majority of trial data for several vaccines provided vaccine titers data against advanced cervical cancer precursors (CIN 2, CIN 3 and adenocarcinoma in situ) and persistent HPV infection. **Figure 4A** showed the HPV genotypes contained in the three prophylactic vaccines.

#### 4.1.1 Gardasil^®^4

In 2006, Merck of the United States available the world’s first quadrivalent vaccine for the prevention of HPV infection. Gardasil^®^4 is an injectable suspension expressed in *Saccharomyces cerevisiae*, is a self-assembled virus-like particle (VLP) composed of purified virus C-terminal truncated L1 protein (the main HPV capsid protein) which is produced by recombinant DNA technology, and it is supplemented with amorphous aluminum hydroxyphosphate sulfate (AAHS) as an adjuvant (Mohsen et al., 2017). It can prevent anal and cervical cancers (70%) caused by HPV 16/18, and genital warts (90%) caused by HPV 6/11 (Flogging Gardasil, 2007). This vaccine provides a high level of vaccine protection for women aged between 15-45 and men aged between16-26.

Gardasil^®^4 has undergone more than 10 clinical trials before marketing, and obtained satisfactory effectiveness, with a prevention rate of 98% for high-grade cervical lesions and protection of non-cervix parts, proving that the vaccines have 100% effectiveness in vulvar and vaginal diseases related to HPV (Quadrivalent Vaccine Against Human Papillomavirus to Prevent High-Grade Cervical Lesions, 2007; Garland et al., 2007). Reports of vaccines effectiveness in different regions claimed that within 6 years after vaccination of Gardasil^®^4, compared with women who were not vaccinated, the number of vaccinated Australian women aged between 18-24 who were infected with the epidemic HPV 6/11/16/18 was dramatically dropped by 86%. Among the women aged between 12-26 who were vaccinated, the low-grade and high-grade cervical lesions declined 34% and 47%, respectively (Garland et al., 2016). In addition, the same trend was observed in the United States, where the prevalence of 4vHPV was decreased by 64% in women aged 14-19 and 34% in women aged 20-24 within six years after vaccination (Markowitz et al., 2016). Although most efficacy trials have been conducted in women aged 15-26, according to the results of the immunobridging studies, the vaccines were initially approved for use in women and men aged 9 to 25 years. In this study, the average HPV 16 antibody titers of girls and boys aged 10-15 years one month after the third vaccination were twice the titers of women aged 16-23 years (Herrero et al., 2015). These data indicated that Gardasil^®^4 has a good population and prophylaxis effect.

#### 4.1.2 Cervarix™

The bivalent HPV vaccine Cervarix™ (GlaxoSmithKline, GSK) was approved by the European Medicines Agency (EMA) in September 2007 and by the FDA in October 2009. Cervarix™ is effective and protective against the most common oncogenic genotypes of HPV (HPV16 and HPV18) (de Sanjose et al., 2010). Because it does not contain other types, it cannot prevent and protect people against lesions such as condyloma acuminatum. Cervarix™ contains HPV16/18 VLPs and AS04 as adjuvants. AS04 includes monophosphate lipid A (MPL) and aluminum hydroxide. Monophosphoryl lipid A (MPL) is a detoxifying bacterial lipopolysaccharide and a toll-like receptor 4 agonist used to activate innate and adaptive immune responses (Mitchell and Casella, 2017).

Clinical data in 2009 showed that Cervarix™ had a 98.1% protection rate against HPV16/18-related CIN2 and CIN2+ (Paavonen et al., 2009). Data in 2017 showed that vaccines continued to prevent infection, cytological abnormalities and lesions related to HPV16/18 CIN1+ in women over 25 years old who received the HPV16/18 vaccines and were followed up for 7 years (Wheeler et al., 2016). Cervarix™ was 100% effective against incidental HPV16/18-related CIN2+/CIN3 in year 11 in the long-term trial designed to assess the effectiveness of the bivalent vaccine against HPV16/18 infection in women aged 18-25 years, and the cumulative efficiency were 97.4% and 94.9%, respectively (Porras et al., 2020). The quadrivalent vaccine was administered at a wide age range, and so did the Cervarix™, which induces a strong immune response in women under 55 years of age. According to the report, although the antibody titer in women aged 26-55 was lower than that in young women (15-25 years old), the antibody titer of HPV 16/18 was still several times higher than that produced by natural infection after 4 years (Schwarz et al., 2011). Non-cervical protection was demonstrated in the Costa Rican vaccine trial, which showed an 84% reduction in anal infections of HPV16/18 in young women four years after vaccination (Kreimer et al., 2011). In vaccine trials involving about 1,000 women, HPV16/18 vulvar infections were reduced by 50% (Lang Kuhs et al., 2014). Although the bivalent vaccines have less protection for two HPV types than the quadrivalent vaccines, they have a better preventive effect on advanced lesions.

#### 4.1.3 Gardasil^®^9

In December 2014, the nonavalent vaccine Gardasil^®^9 was available by Merck in the United States and the Advisory Committee on Immunization Practice (ACIP) recommended Gardasil^®^9 as one of the three HPV vaccines allowed and set a routine vaccination in February 2015 (Petrosky et al., 2015). Gardasil^®^9 follows the expression system and production method of Gardasil^®^4, but it covers the other five HPV types and can provide protection against HPV Genotypes 6, 11, 16, 18, 31, 33, 45, 53 and 58. As a result, Gardasil^®^9 can prevents 90% of cervical cancers (Cheng et al., 2020).

An analysis of the efficacy and immunogenicity of the nonavalent vaccine in more than 14,000 women aged 16-26 years in a phase III trial showed non-inferior immune responses to HPV 6, 11, 16, and 18 compared with the quadrivalent vaccine. Efficacy of the vaccine against CIN2+, VIN2+, vulvar and vaginal lesions caused by HPV 31, 33, 45, 52 and 58 was 96.7% (95% CI 80.9-99.8) (Joura et al., 2015). Clinical data reported in 2017 showed that Gardasil^®^9 was 97.4% protective against high-grade cervical, vulvar, and vaginal lesions in women aged 16-26 (Huh et al., 2017). However, due to differences in the prevalence and distribution of specific types of HPV, the protection provided by vaccines to people in different regions and different races is slightly different. The protection rates are about 87.7% in Asia, 91.7% in Africa, 92% in North America, 90.9% in Europe, and 86.5% in Australia (Zhai and Tumban, 2016).

#### 4.1.4 The Dosage and Safety of the HPV Prophylactic Vaccines

The Costa Rica bivalent vaccine trial found that 100% of women, irrespective of the number of doses, remained seropositive for HPV16/18 at 4 years regardless of dose, while women receiving two doses tested 6 months apart had antibody titers no worse than the three-dose group, and antibody concentrations in the one-dose group remained stable over 4 years (Kreimer et al., 2011; Safaeian et al., 2013). Studies have reported that the immunogenicity of the two-dose bivalent and quadrivalent vaccines was no worse in girls aged 9-14 years than in women aged 15-26 years who received three doses of the vaccine (Dobson et al., 2013; Lazcano-Ponce et al., 2014). With the introduction of the nonavalent vaccine, three additional doses of nonavalent vaccines were given to those previously vaccinated with quadrivalent vaccines, but the clinical significance of the additional five antibody titers found to be lower is not yet known. Currently, the World Health Organization (WHO) endorses a vaccination schedule of two doses of vaccine for boys and girls aged 9-14 years and three doses for those aged 15 years or older, with the decision on the type of vaccine to be made by the patient on an individual basis (Dilley et al., 2020).

The pre-licensure trials included more than 20,000 women aged 9-26 years who received the quadrivalent vaccine and more than 30,000 women aged at least 10 years who received the bivalent vaccine. The most common reaction to both vaccine injection sites was mild pain, while serious adverse events and even death did not differ from the control group and were not clearly determined to be vaccine-related (Block et al., 2010; Macartney et al., 2013; Angelo et al., 2014). The US Vaccine Adverse Events Reporting System showed that for quadrivalent vaccines, post-vaccination syncope was one of the most common adverse events, while the same occurred in the UK and the Netherlands where bivalent vaccines were introduced, but none of the final causes were attributed to the vaccine, thus providing confidence in the safety of the HPV vaccine (Slade et al., 2009; Angelo et al., 2014).

### 4.2 Future Development Direction of HPV Prophylactic Vaccines

There is a high degree of clinical attention to prophylactic vaccines, and many clinical trials have been conducted. **Figures 4B, C** shows the progress of prophylactic vaccines in the clinical (Data from ClinicalTrials.gov). Not only the three common vaccines, but also the current status of vaccines ranging from 1v to 11v, which are designed differently from the expression system and the number of subtypes protected against HPV, are described. The three currently licensed prophylactic vaccines were all developed based on multiple types of mixed L1 VLPs that induce strong immunogenicity and produce high levels of antibody titers (Stanley et al., 2021). However, from the clinical trials described above and the long-term follow-up, it appears that the antibody titer decreases significantly over time and varies greatly between individuals. In addition to booster immunization in a given year, the solution is to develop new adjuvants to enhance the immunogenicity and prolong the persistence of immunity (Huber et al., 2021). From this starting point, in 2019, a research team from Xiamen University, in collaboration with GSK, is developing a new generation of HPV vaccine using GSK’s novel adjuvant. For their expression systems, the three vaccines currently licensed use insect or *Saccharomyces cerevisiae* expression system. However, their production processes are complex and there are major problems in the expression and purification of VLPs, leading to expensive vaccine costs. Therefore, starting from the problem of reducing costs and increasing the production efficiency of L1 VLP, the development of expression systems can be mainly focused on lower-cost *Escherichia coli* (*E. coli*) or other alternative types of yeast, such as *Hansenula polymorpha* (*H. polymorpha*) and *Pichia pastoris* (*P. pastoris*). We also expect that more expression systems will be developed in the future to facilitate large-scale processing and culturing of products.

The key feature of current prophylactic vaccines using L1 VLPs is their ability to form VLPs spontaneously and with high immunogenicity (Joura et al., 2015). However, the L1 VLP vaccine only provides protection against a single HPV type, which has led to interest in exploring L2 vaccines with broad-spectrum protection. L2 is highly conserved among HPV types and does not spontaneously form VLPs, also resulting in L2-induced neutralizing antibody titers that are typically much lower than those induced by homologous L1 VLPs (Wang and Roden, 2013). This was demonstrated in early studies where it was found that recombinantly expressed L2 could act as an immunogen against papillomavirus infection, but its ability to induce neutralizing antibodies was poor (Lin et al., 1992). Therefore, in the development of L2 vaccine, improving its immunogenicity is the biggest challenge. One approach is to fuse L2 to immunostimulatory molecules, and in one study, the use of bacterial flagellin (Fla), a ligand for toll like receptor 5, fused to L2 and used in conjunction with aluminum adjuvant provided durable immunity in an animal model (Kalnin et al., 2017). Of course, the expression of peptides in carrier proteins and VLP forms is also a common way to enhance immunogenicity. A phage VLP based vaccine has been reported, which displays short L2 peptide and induces high titer and a wide range of protective antibody responses (Tumban et al., 2013). The use of highly potent adjuvants is arguably the easiest approach to think of, such as aluminum hydroxide/monophosphoryl lipid A (Alum/MPL) adjuvants (Schellenbacher et al., 2009). It is not a bad idea to make a bold prediction about the future direction of L2 vaccines. In the future, antigen combinations will increase, such as L2 chimeric L1 VLP, L2 combined with E6/E7 for prevention and treatment of a full range of conditions, as well as a move from the current monotypic to multitypic L2 fusions to provide prevention against most HPV subtypes.

To summarize, the development of L1 VLP is focused on reducing costs and improving the durable immunity of the vaccine. The development of L2 vaccines is dedicated to enhancing their immunogenicity and to combine with other antigens or drugs to achieve broad prophylactic effects.

## 5 Research Progress of HPV Therapeutic Vaccines

The mechanism of HPV prophylactic vaccines is to cause the body’s humoral immunity to induce the generation of neutralizing antibodies to prevent HPV infection. However, the HPV preventive vaccine does not provide effective treatment for an already infected organism. Besides, with the integration of the virus genome into the host genome, many early genes (E1, E2, E4, E5) and late genes (L1, L2) are missing, making prophylactic vaccines ineffective against HPV-related precancerous lesions and cancers (zur Hausen, 2002).

**Figure 5:**
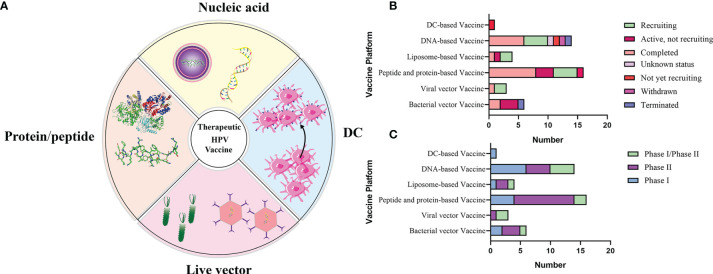
**(A)** Schematic diagram of several major types of therapeutic vaccines. **(B, C)** Status and phase of therapeutic vaccines (Data from ClinicalTrials.gov). Most of the clinical trials we found were in Phase I/II, and few of the completed clinical trials have progressed to the next phase, which has resulted in no therapeutic vaccines being available at this time.

There are mainly four categories of HPV treatment vaccine such as live vector-based vaccines, peptide and protein-based vaccines, nucleic acid-based vaccines and whole cell vaccines, and their demonstration diagram is shown in **Figure 5A**. The working mechanism of these vaccines is to delivery target antigen to antigen presenting cells (APC), and then activate HPV specific cytotoxic CD8^+^T lymphocyte (CTL) responses, and the helper CD4^+^T lymphocyte (Th) responses. Oncoproteins E6 and E7 are important transforming proteins for the occurrence and maintenance of HPV-related malignant tumors, the two most important target antigens for HPV therapeutic vaccines, and most widely used in existing development schemes. Vaccines designed based on E2 antigen are also of great significance. E2 is a negative regulator of E6 and E7, and its expression in precancerous lesions (mainly primary precancerous lesions) is higher than E6 and E7 so that vaccines designed for E2 antigen are mainly used for the treatment of precancerous lesions, genital warts (condyloma acuminatum), and other diseases (Smith et al., 2014).

### 5.1 Live Vector-Based Vaccines

Live vector-based vaccines can be divided into two main categories: bacterial vector-based vaccines and viral vector-based vaccines. These vaccines use attenuated vector carriers encoding genes for HPV-specific antigens (E6, E7) to replicate in host cells and induce an immune response against HPV (Yang et al., 2016). It has high immunogenicity and can cause strong humoral and cellular immunity, but it will also cause corresponding problems such as potential safety risks for people with low immunity (Ma et al., 2012). What’s worse, it is possible that the body’s immune response to the vector itself is stronger than its immune response to the corresponding antigen, which is another barrier to using live vector-based vaccines (Wang et al., 2020).

#### 5.1.1 Bacterial Vector-Based Vaccines

Bacterial vectors such as Listeria monocytogenes (Lm), Lactobacillus casei (L.casei), and Salmonella (SE) have been used in clinical trial vaccines, but the development of bacterial vector-based vaccines has been limited due to safety and effectiveness problems. However, such vaccines are undoubtedly indispensable in the development of therapeutic vaccines.

ADXS11-001, also known as ADXS-HPV or Lm-LLO-E7, is a live, attenuated bacterial vector-based vaccine based on Listeria monocytogenes (Lm). The antigen is composed of human HPV16 E7 fused with a truncated non-hemolytic LLO fragment and the first clinical study was in 2009 (Maciag et al., 2009). Listeria monocytogenes (Lm) is a gram-positive facultative intracellular bacterium that interacts with the receptor protein on the host cell to enter the cell, but what makes it different from other bacteria is that it uses the pore-forming toxin listeriolysin O (LLO) and phospholipase C (PLC), and enables them to escape from the host cell phagosome into the cytoplasm of the host cell (Chen and Wu, 1998; Guirnalda et al., 2012). Two MHC pathways are generated to activate the adaptive response. First for bacteria that cannot escape phagosomes, the MHC class II pathway is activated to stimulate CD4^+^T cells, and then for those bacteria that successfully escape the host cell phagosome, the MHC class I pathway is used to extract polypeptides from bacterial antigens and then make them presented on the surface of host cells, where CD8^+^T cells are activated (Cory and Chu, 2014). The phase II clinical trial evaluated the safety and efficacy of ADXS11-001 in patients with relapsed/refractory cervical cancer after chemotherapy and/or radiotherapy. According to the analysis of the trial results, the tolerability of ADXS11-001 monotherapy group is excellent and there are more adverse reactions (AEs) in the combined cisplatin treatment group. The median overall survival (OS) was comparable between the monotherapy group and the combination group (ADXS11-001: 8.28 months; 95% confidence interval [CI], 5.85–10.5 months; ADXS11-001 + cisplatin: 8.78 months; 95% [CI], 7.4–13.3 months). For the total OS rate of the two groups, 34.9% (38/109) patients achieved an OS of 12 months, and 24.8% (27/109) of patients achieved an OS of 18 months. It is an encouraging result that warrants further study (Basu et al., 2018).

GLBL101c vaccine is produced by heat-attenuated recombinant Lactobacillus casei (L.casei) expressing mutant HPV16 E7, and it is a vaccine for oral therapy. Previous Phase I/IIa clinical trials demonstrated that oral GLBL101c vaccines can cause the regression of HPV16 E7-associated CIN 3 and the regression rate from CIN 3 to CIN 2 was 80% after 9 weeks of treatment (Kawana et al., 2014). A recent trial investigated the efficacy and adverse reactions of GLBL101c in patients with CIN 2, the results showed that there were no serious adverse reactions. The CR of GLBL101c and placebo groups were 11% (2/19 cases) and 0% (0/19 cases) respectively, and the effective rate of CR+PR, was 22% (Ikeda et al., 2021). The results show that the clinical efficacy is not ideal and it is necessary to develop new protocols.

#### 5.1.2 Viral Vector-Based Vaccines

Replication-deficient viral vectors are attractive vectors, and widely cited viral vectors include adenoviruses, adeno-associated viruses, alphaviruses, lentiviruses and vaccinia viruses (Liu et al., 2000; Gomez-Gutierrez et al., 2007; Zurkova et al., 2009; Ip et al., 2015). Adenovirus-based (Ad) technology is the most advanced genetic vaccination technology. The most advantageous feature of Ad vectors is their ability to induce a strong systemic T-lymphocyte response and high serum antibodies following intramuscular (IM) immunization (Barouch et al., 2011). As a vaccine vector, Ad5 is the most common human serotype and the most widely used serotype. Vector technology based on multiple Ad serotypes has achieved good results in vaccination of various diseases (Ewer et al., 2016). There is a study based on HPV therapeutic vaccines of E1/E3 deletion replication-deficient Ad26 and Ad35, expressing HPV16 E6/E7, the fusion protein. Immunization of mice elicited a strong E6/E7 specific CD8^+^T cell response to secrete multifunctional cytokines, but no specific CD4^+^T cell response was detected (Çuburu et al., 2018). It is also based on another therapeutic vaccine of Ad26 and Ad35, expressing the early protein E2/E6/E7 of HPV16/18, which aims to treat diseases of HPV infection at all stages. The coding sequence of antigen E6 was divided into 3 segments and E7 was divided into 2 segments, and then recombined. The E2 antigen and the N-terminal fusion to form two antigens (HPV16E2SH: E2+E6_1-44_+E7_35-98_+E6_92-157_+E7_1-59_+E6_21-115_; HPV18E2SH: E2+E6_1-39_+E7_24-104_+E6_80-156_+E7_1-45_+E6_15-103_) (Khan et al., 2017). Good results were obtained in both the immunogenicity in mice and the TC-1 tumor model.

Vaccinia virus is a double-stranded DNA virus with a large and stable genome that can express a large number of antigens. Vaccinia virus has been widely used as an immunogen and has a good tolerance (Borysiewicz et al., 1996). TA-HPV is a recombinant vaccinia virus vaccine expressing HPV16/18 E6 and E7 proteins. In a very early clinical phase I/II trial of 8 patients with advanced cervical cancer, HPV was found in 3 patients (3/8) specific antibody response, and HPV specific cytotoxic T lymphocyte response was detected in 1 patient (1/3). In the latter TA-HPV and cisplatin combined treatment experiment, and in the mouse TC-1 tumor model produce a stronger E7-specific CD8^+^T lymphocyte response (Lee et al., 2013). Recombinant vaccinia virus MVA E2 is a vaccinia virus Ankara (MVA) containing bovine papillomavirus E2 protein. As an E6/E7 expression inhibitor, E2 introduced into the host may inhibit the activity of E6 and E7, thereby treating precancerous lesions and even inhibiting cancer. MVA E2 has been proven to prevent the growth of human tumors in mice and induce tumor regression in tumor-bearing rabbits (Rosales et al., 2000). MVA E2 was used to treat HPV-induced anogenital intraepithelial lesions in a phase III clinical trial in 2014 involving 1356 patients (male and female), resulting in an overall efficacy of approximately 90% in the treatment of CIN lesions, with all men showing complete eradication (Rosales et al., 2014). In the latest I/II clinical trial evaluating the possibility of MVA E2 therapeutic vaccines reducing the possibility of recurrence of respiratory papillomatosis (RRP), 13 cases (13/29, 44.8%) of lesions were completely eliminated, and 16 cases (16/29, 55.2%) of lesions recurred 6 to 18 months after treatment. Subsequently, after a second round of MVA E2 therapy, no new recurrence symptoms were observed, indicating that MVA E2 vaccines have a good potential for complete regression of RRP lesions (Cabo Beltran and Rosales Ledezma, 2019). The TG4001 vaccine expresses HPV16 E6/E7, and in a trial evaluating safety and efficacy in patients with (CIN) 2/3, HPV 16 mRNA clearance was associated with CIN 2/3 cytologic and colposcopic regression in 7 of 10 patients (Brun et al., 2011). These promising data warrant further development of TG4001 in the treatment of CIN 2/3. Vvax001, a therapeutic alphavirus-based cancer vaccine expressing HPV16 E6/E7, was evaluated for immunological activity, safety and tolerability in a first-in-human phase I trial. The results were that immunization with Vvax001 was safe and well tolerated, with only mild injection site reactions and resulted in CD4+ and CD8+ T cell responses against E6 and E7 antigens (Komdeur et al., 2021).

Replicating viral vector vaccines also hold good promise for the treatment of HPV-associated cancers, such as foamy viruses (FVs). Replication-competent FVs can trigger immune signaling and integrate into the host genome, resulting in sustained antigen expression and a robust immune response. One study explored the feline foam virus (FFV) protein as a scaffold for *in vitro* therapeutic B and T cell epitope delivery, and immunizing mice with the T cell epitope peptide E7_49-57_ of HPV16 E7 attached to its expression vector protected mice from HPV16-transformed tumor cells (Lei et al., 2015).

### 5.2 Peptide and Protein-Based Vaccines

Such vaccines present antigens in the form of peptides or proteins, which are acquired, processed, and presented by DC to activate MHC I or II molecular pathways to stimulate the immune response of CD8^+^T or CD4^+^T cells (Ma et al., 2012). Peptide vaccines are divided into synthetic long peptides (SLP) and specific epitope (short) peptides. Short peptides are MHC-specific and need to match a specific type of human leukocyte antigen (HLA) while long peptide vaccines and protein vaccines are rich in CD4^+^T and CD8^+^T cell epitopes, which can avoid the limitations of MHC restriction (Paris et al., 2012; Lee et al., 2016). Protein and peptide vaccines are safe and stable, but the poor immunogenicity of protein vaccines is a major drawback in their development and the focus on the MHC II pathway of presentation resulting in a less than robust CTL response. Improvements in protein vaccines can be achieved by adding adjuvants or immunostimulatory molecules to increase endogenous processing and enhance the MHC I pathway (Valdez Graham et al., 2000).

ISA 101, a long peptide HPV16 vaccine, synthesized from 9 overlapping E6 long peptides and 4 overlapping E7 long peptides, dissolved in dimethyl sulfoxide in 20 mM phosphate buffered saline (pH 7.5) and emulsified with Montanide ISA-51 (Kenter et al., 2009). In phase II, ISA 101 combined with Nivolumab, an anti-PD-1 immune checkpoint antibody, was evaluated in patients with untreatable HPV16-positive cancer (Massarelli et al., 2019). Compared with the use of anti-PD-1 antibody alone, this regimen achieved an overall response rate of 33% and a median overall survival of 17.5 months, suggesting the need for further research. In another clinical trial of 77 patients with advanced, recurrent or metastatic cervical cancer receiving both the ISA101 vaccine and standard chemotherapy with carboplatin and paclitaxel, tumor regression was observed in 43% of patients (72 evaluable patients), and all patients had a type 1 T-cell response to the vaccine, suggesting that chemoimmunotherapy can be used to enable effective treatment of patients with advanced cancer (Melief et al., 2020).

A study used mHSP110 (heat shock protein) as an immune adjuvant to enhance the immune response to HPV16 E7-derived CTL epitope E7 (Garland et al., 2007; Quadrivalent Vaccine Against Human Papillomavirus to Prevent High-Grade Cervical Lesions, 2007; Paavonen et al., 2009; de Sanjose et al., 2010; Herrero et al., 2015; Garland et al., 2016; Markowitz et al., 2016; Wheeler et al., 2016; Mitchell and Casella, 2017) in a mouse model. HSP110 has a high binding affinity to proteins and can enhance immunogenicity of protein antigens. They developed the mHSP110-E7 (Garland et al., 2007; Quadrivalent Vaccine Against Human Papillomavirus to Prevent High-Grade Cervical Lesions, 2007; Paavonen et al., 2009; de Sanjose et al., 2010; Herrero et al., 2015; Garland et al., 2016; Markowitz et al., 2016; Wheeler et al., 2016; Mitchell and Casella, 2017) fusion protein and used FITC labeled E7 (Garland et al., 2007; Quadrivalent Vaccine Against Human Papillomavirus to Prevent High-Grade Cervical Lesions, 2007; Paavonen et al., 2009; de Sanjose et al., 2010; Herrero et al., 2015; Garland et al., 2016; Markowitz et al., 2016; Wheeler et al., 2016; Mitchell and Casella, 2017) as a tracer to prove that mHSP110 formed a complex with E7 (Garland et al., 2007; Quadrivalent Vaccine Against Human Papillomavirus to Prevent High-Grade Cervical Lesions, 2007; Paavonen et al., 2009; de Sanjose et al., 2010; Herrero et al., 2015; Garland et al., 2016; Markowitz et al., 2016; Wheeler et al., 2016; Mitchell and Casella, 2017). Then immunizing mice can induce strong CTL response and can protect mice from tumor attack, significantly inhibiting the growth of established tumors in the anti-tumor test and prolonging the survival time of tumor-bearing animals (Ren et al., 2010).

Fibronectin additional domain A (EDA) is a toll-like receptor 4 (TLR 4) protein agonist that targets antigen to DCs *in vivo* and induces maturation by TLR4 binding, better delivering the treated short peptide to naïve T cells (Mansilla et al., 2012). The full-length E7 protein of HPV16/18 and the extra domain A of human fibronectin (hEDA) were fused to form a bivalent recombinant protein, combined with Poly-IC (polyinosinic-polycytidylic acid) and Poly-ICLC (a synthetic complex of carboxymethylcellulose, polyinosinic-polycytidylic acid, and poly-L-lysine double-stranded RNA) adjuvants to evaluate the effect, immunogenicity and potential therapeutic activity of HPV16 TC-1 tumors *in situ* in subcutaneous or genital. Results showed that the vaccine induced E7 specific cytotoxic T-lymphocyte (CTL) response and eliminated established tumors, with some groups achieving 100% anti-tumor effects when combined with adjuvant (Arribillaga et al., 2020). This is a very exciting result and also holds promise for good clinical outcomes.

In conclusion, hEDA-HPVE7-16/18+Poly-ICLC is a promising regimen for the treatment of cancer and the refinement of its protocol to enable it to improve the benefits of clinical treatment. A few points of improvement are analyzed, one being the future consideration of the effect of tumor suppressive environment on vaccine efficacy as proposed in its article itself, meaning creating an environment closer to tumor growth *in vivo*. The second is the improvement of its experimental design, which on the one hand can supplement the treatment data of the vaccine on the experimental group with larger tumors, which can reflect the treatment level of the vaccine on patients with advanced disease. On the other hand, the dose of the vaccine is an important factor in immune efficacy, and high doses of vaccine injections may cause adverse reactions, which should be documented as they arise.

### 5.3 Nucleic Acid-Based Vaccines

#### 5.3.1 DNA Vaccines

DNA vaccines have become an attractive immunotherapy method for the treatment of cancer due to their advantages such as simplicity, stability, and effective antigen-specific immunotherapy. DNA vaccines are based on bacterial plasmids that encode antigens driven by high-efficiency eukaryotic promoters and the effective DNA vaccines must enter the nucleus after injection to induce expression of antigens which are delivered by MHC class I molecules to activate the immune system (Rajcáni et al., 2005; Li and Petrovsky, 2016). Unlike live vector-based vaccines, DNA vaccines are relatively safe and do not produce antibodies against the vector in the body and can be used through repeated vaccinations to boost immunity (Yang et al., 2014). The inability of exposed DNA to amplify on its own, resulting in poor immunogenicity in the body, is one of the main disadvantages of DNA vaccines. One of the main drawbacks of the DNA vaccines is their poor immunogenicity. In order to improve immunogenicity, a series of methods such as electroporation of DC cells, the use of immunomodulators (such as strong adjuvants) and other immunostimulants (such as cytokines and costimulatory molecules) are used in vaccine design (Vici et al., 2016; Afrough et al., 2019).

A clinical trial evaluated the safety, effectiveness and immunogenicity of the DNA vaccines of pNGVL4a-CRT/E7 in 16 HPV-related CIN 2/3 patients (Alvarez et al., 2016). The DNA vaccine consists of the pNGVL4a expression vector, which contains the HPV 16 E7 coding sequence linked to calreticulin (CRT) (Cheng et al., 2001). 32 cases of HPV16 CIN2/3 patients were vaccinated by epidermal administration, intramuscular injection, or direct intrauterine injection, and 22 (69%) patients experienced adverse vaccine-related reactions, and 8 of 27 (30%) had histological regression of CIN 1 or below. The corresponding data showed that a strong immune response was induced and more CD8^+^T-lymphocyte responses occurred (Alvarez et al., 2016).

A recent clinical trial explored the therapeutic effect of the therapeutic DNA vaccine GX-188e on the regression of cervical intraepithelial neoplasia (CIN3) (Choi et al., 2020). GX-188e is composed of tissue plasminogen activator signal sequence, FMS-like tyrosine kinase 3 ligand and recombinant HPV 16/18 E6/E7 gene (Kim et al., 2014). As a result, 52% of V7 patients and 67% of V8 patients experienced histopathological regression, 73% (V7) and 77% (V8) of histological regression patients showed HPV clearance (Choi et al., 2020). This demonstrates that vaccination with GX-188E vaccine induces strong cellular immunity to clear HPV histological lesions. In another trial of AMV 002 vaccination, results showed that the AMV 002 vaccine was well tolerated at all dose levels and enhanced specific immunity to tumor-associated antigens in previously treated patients (Chandra et al., 2021).

#### 5.3.2 mRNA Vaccines

The mRNA vaccine, the current popular form of vaccines, has been widely proven to be a promising therapeutic strategy in immunotherapy. In 1989, Malone et al, demonstrated that mRNA could be successfully transfected and expressed in various eukaryotic cells by encapsulation of a cationic lipid (N-[1-(2,3-dioleyloxy)propyl]-N,N,N-trimethylammonium chloride (DOTMA)) (Malone et al., 1989). Subsequently, in 1990, mRNA transcribed *in vitro* was fully expressed in mouse skeletal muscle cells, and the successful expression of mRNA *in vitro* for the first time proved the feasibility of developing a mRNA vaccine (Wolff et al., 1990). The mRNA structure includes a 5’cap structure, 5’ and 3’UTR structures, a coding sequence, and a 3’poly A tail (Maruggi et al., 2019). A large number of studies have shown that mRNA is unintegrated (safe) and that the new generation of self-amplifying mRNA vaccines (saRNA vaccines) have a high capacity for autonomous replication. Then the self-replicating RNA viral vectors have high expression levels, and have TLR 7/8 ligand activity, which is a natural adjuvant that can induce a strong immune response (Diebold et al., 2004; Brito et al., 2015). Compared with DNA vaccines, the important reasons that determines the slow development of mRNA vaccines are their poor stability and low delivery efficiency. Therefore, mRNA is often packaged into the body by delivery vectors, including DC vectors, protamine, cationic lipid delivery systems and polymer materials (Lungwitz et al., 2005; Christensen et al., 2009; Kallen et al., 2013; Pardi et al., 2018).

There are few reports on HPV mRNA vaccines. A new study designed that the mRNA expressing the antigen HPV16 E7 is encapsulated in liposome preparations to form RNA-lipid complexes (RNA-LPX) and the immunogenicity of the vaccine is evaluated by intravenous injection, inducing a strong antigen-specific effect and memory CD8^+^T cell response in mice (Grunwitz et al., 2019). With the outbreak of the new coronavirus, mRNA vaccine research has been pushed to a higher degree, and the development prospects are also bright.

In conclusion, nucleic acid vaccines have not been studied much in the field of HPV and have produced different results in animal experiments. Currently, mRNA vaccines are becoming more popular in the context of the epidemic thanks to their safety and good efficacy.

### 5.4 Whole Cell Vaccines

#### 5.4.1 DC Vaccines

Dendritic cells (DCs) are the strongest and most effective APCs in presenting antigens and play an important role in immune regulation. It has a strong ability to acquire and process antigens for presentation to T lymphocytes *in vivo* and *in vitro*, many evidences have confirmed the ability of monocyte-derived DCs to stimulate naïve CD4^+^ T and CD8^+^ T lymphocytes *in vitro* and *in vivo (* Santin et al., 1999; Santin et al., 2006). Moreover, DC also acts as a natural adjuvant to enhance the immunogenicity of vaccines (Santin et al., 2005). There are two methods for preparing HPV vaccines with DCs as the core. One is to culture DCs *in vitro*, and then to stimulate DCs with HPV E6/E7 antigen. And another is that DCs stably transfected *in vitro* with a vector expressing HPV antigen, and then DC vaccines adoptively transferred into the patient, presenting the antigen to naïve T cells and inducing CTL response (Wang et al., 2000; Peng et al., 2005). The application of TOLL-like receptor agonists to promote the maturation of DC is also widely used in the treatment of DC vaccines. Toll-like receptor (TLR) is a part of DC cell pattern recognition receptor, and DC cell pattern recognition receptor also has C-type lectin receptor (CLR), Nucleotide-binding oligomerization domain-like receptor (NLR) and retinoic acid induced gene like receptor (RLR) (Takeuchi and Akira, 2010; Kawasaki et al., 2011). TLR ligand can induce DC cell phenotype and functional maturation, and regulate cell metabolism and lifespan (Chen et al., 2007; Li et al., 2010).

The safety and immunogenicity of the HPV16/18 E7 antigen and keyhole limpet hemocyanin (KLH) pulsed mature dendritic cell (DC) vaccination were evaluated for patients with stage IB or IIA cervical cancer (Santin et al., 2008). Three doses (low, medium, and high) were designed to be administered every 21 days (5 times in total). Patients receiving DC vaccines showed good tolerability and no significant toxic and side effects, and significantly increased E7 and KLH-specific CD4^+^T expression after vaccination.

Camelid-derived single-domain antibody fragments (nanobodies or VHHs) recognize cell surface proteins on antigen-presenting cells (APCs) and can serve as targeted delivery vehicles for antigens attached to them. Therefore, one study targeted VHH+CD11b-E7_49-57_ to DC2.4 cells, and vaccine immunization of mice resulted in the production of more CD8 tumor-infiltrating lymphocytes in HPV-tumor-bearing mice (Woodham et al., 2018).

DC vaccines also have limitations. Firstly, due to the limitation of preparation technology, the quantity and quality of extracted DC cannot be guaranteed. Other, it is difficult to produce on a large scale, and different processes may lead to inconsistent vaccine quality. So, there are still a lot of hurdles to overcome for DC vaccines development.

#### 5.4.2 Tumor Vaccines

Each cancer has a large number of potentially tumorigenic antigens, so vaccinating whole tumor cells is the best strategy to include all potentially relevant antigens. Moreover, this vaccine approach circumvents the limitations of the major histocompatibility complex (MHC) and does not require epitope identification on a patient-tailored basis (Tagliamonte et al., 2014). The efficacy of this approach has been evaluated over the years in clinical trials for different tumors, including lung, colorectal, melanoma renal cell carcinoma and prostate cancer. Because HPV is a well-known tumor-specific antigen, tumor cell-based vaccines may not be the most practical immunotherapy for HPV-associated cancers, and fewer trials have been done to evaluate the true utility of this type of vaccine for HPV-associated cancers.

### 5.5 Improvement of HPV Therapeutic Vaccines

The role of therapeutic vaccines is to enhance adaptive T cell immunity by initiating naïve T cells to produce cytotoxic T lymphocytes (CTL) that target HPV infected cells, inducing CD4^+^T cells to produce necessary cytokines, and enhancing antigen presenting cells (APC) (Lee et al., 2016). At the current stage, all therapeutic vaccines tested in clinical trials are reported to be safe and well tolerated. **Table 2** summarizes the current therapeutic vaccines in the clinical stage, and the progress diagram is shown in **Figures 5B, C**. However, many vaccines that have entered clinical phase III trials have not yet been announced for marketing because of their lower expected clinical effects. This is also related to the fact that these vaccines have shown success in animal models but are not effective in human HPV-induced cancers, which highlights the limitations of the currently used preclinical models (Hancock et al., 2018). In fact, a large number of immunosuppressive environments can be observed in the tumor microenvironment, which makes the efficacy of vaccine-induced T cells affected by a variety of immune evasion and immunosuppressive mechanisms to reduce the immune effect. For example, cancer-associated fibroblasts (CAF) have pro-tumorigenic functions and cause immune evasion of tumors through several mechanisms. CD8^+^T cell exclusion was present in CAF-rich human tumors. One study found poor immune effects of tumors with CAF by injecting CAF mixed with target cells into mice to form a state similar to the human tumor environment and then performing immunotherapy (Ford et al., 2020). Therefore, the impact of tumor environment on vaccine efficacy is important, for example, we need to establish more *in situ* tumors to provide for preclinical trials including cervical, head and neck *in situ* tumors.

**Table 2:** Summary of clinical HPV therapeutic vaccines.

Vaccine Platform	Vaccine	Antigen	Conditions	Phase/NCT Number	Study Start	Status
Bacterial vector Vaccine	ADXS11-001	HPV16 E7	EAs,UCC	Phase II/NCT01266460	May 23,2011	Completed
			OC	Phase I/NCT01598792	February 2012	Terminated
			AC,RC	Phase II/NCT02399813	September 2015	Completed
			UCC,SCCHN	Phase I/Phase II NCT02291055	April 2015	Active, not recruiting
			SCCHN	Phase II/NCT02002182	December 2013	Active, not recruiting
	Ad/MG1-E6E7	HPV16/18 E6/E7	HPV-Associated Cancers	Phase I/NCT03618953	June 21,2018	Active, not recruiting
Viral vector Vaccine	TG4001	HPV16 E6/E7	UCC,ASCC	Phase I/Phase II NCT03260023	September 11,2017	Recruiting
	TA-HPV	HPV16/18 E6/E7	UCC	Phase II/NCT00002916	November 1996	Completed
	PRGN-2009	HPV16/18 E6/E7	UCC,OC,RC,AC	Phase I/Phase II NCT04432597	August 11,2020	Recruiting
Peptide and protein-based Vaccine	TVGV-1	HPV16 E7	HSIL	Phase II/NCT02576561	November 2015	Unknown status
	TA-CIN	HPV16 L2/E6/E7	UCC	Phase I/NCT02405221	April 4,2019	Recruiting
	ProCervix	HPV16/18 E7	Genital Infection Viral	Phase II/NCT01957878	December 2013	Completed
	PepCan	HPV16 E6	SCCHN	Phase I/Phase II NCT03821272	November 13,2019	Recruiting
			HSIL	Phase II/NCT02481414	November 30,2015	Active, not recruiting
	ISA101b	HPV16 E6/E7	UCC	Phase I/Phase II NCT02128126	September 2013	Completed
			SCC,SCCHN	Phase II/NCT04369937	July 6,2020	Recruiting
			UCC	Phase II/NCT04646005	June 28,2021	Recruiting
	ISA 101	HPV16 E6/E7	Malignant Neoplasms of Lip Oral Cavity and Pharynx	Phase II/NCT03258008	April 4,2018	Active, not recruiting
			Solid Tumors	Phase II/NCT02426892	December 23,2015	Active, not recruiting
	Human papillomavirus 16 E7 peptide	HPV16 E7	UCC	Phase I/NCT00003977	November 1999	Completed
	human papillomavirus 16 E6/E7 peptide	HPV16 E6/E7	AC,UCC,EC	Phase I/NCT00019110	November 1995	Completed
	SGN-00101	HPV16 E7	RRP	Phase II/NCT00038714	November 2001	Completed
			UCC,CIN III	Phase II/NCT00075569	March 2004	Completed
			UCC,CIN III	Phase II/NCT00054041	June 2004	Completed
	Hespecta	HPV16 E6	Tumors or Premalignant Lesions	Phase I/NCT02821494	March 2015	Completed
Liposome-based Vaccine	PDS0101	HPV16 E6/E7	SCCHN,OPSCC	Phase II/NCT04260126	March 29,2021	Recruiting
			UCC IB3/II	Phase II/NCT04580771	October 14,2020	Recruiting
			CIN I	Phase I/NCT02065973	February 2014	Completed
	DPX-E7	HPV16 E7	SCCHN,UCC,AC	Phase I/Phase II NCT02865135	December 2016	Active, not recruiting
DNA-based Vaccine/Viral vector Vaccine	pNGVL4a-Sig/E7(detox)/HSP70 with TA-HPV	HPV16/18 E6/E7	UCC,CIN III	Phase I/NCT00788164	November 2008	Recruiting
DNA-based Vaccine/ Peptide and protein-based Vaccine	pNGVL4a-Sig/E7(detox)/HSP70 with TA-CIN	HPV16 L2/E6/E7	ASC-US,ASC-H,LSIL	Phase II/NCT03911076	May 22,2019	Recruiting
	pNGVL4aCRTE6E7L2 with TA-CIN	HPV16 L2/E6/E7	ASC-US,LSIL	Phase I/NCT03913117	December 31,2021	Not yet recruiting
DNA-based Vaccine	VGX-3100	HPV16/18 E6/E7	CIN II/III	Phase I/NCT01304524	April 2011	Completed
	pNGVL4a-Sig/E7(detox)/HSP70	HPV16 E7	UCC,CIN II/III	Phase I/Phase II NCT00121173	November 2003	Completed
	pNGVL4aCRTE6E7L2	HPV16 L2/E6/E7	CIN II/III	Phase I/NCT04131413	September 14,2020	Recruiting
	pNGVL4a-CRT/E7(Detox)	HPV16 E7	SCCHN	Phase I/NCT01493154	April 2012	Terminated
			CIN II/III	Phase I/NCT00988559	September 2009	Completed
	INO-3112	HPV16/18 E6/E7	SCCHN	Phase I/Phase II NCT02163057	August 13,2014	Completed
			UCC	Phase I/Phase II NCT02172911	June 6,2014	Completed
			UCC	Phase II/NCT02501278	May 2016	Withdrawn
	GX-188E	HPV16/18 E6/E7	UCC	Phase I/Phase II NCT03444376	May 23,2018	Recruiting
			CIN I	Phase II/NCT02596243	August 2015	Unknown status
			CIN I	Phase II/NCT02139267	July 2014	Completed
DC-based Vaccine	DC Vaccines Targeting HPV E6/E7 Protein	HPV16/18 E6/E7	CIN I/II	Phase I/NCT03870113	April 1,2019	Not yet recruiting

The above is a reflection on the importance of the tumor environment, followed by an in-depth discussion on vaccine improvement. One is to improve the immunogenicity of vaccines at the current research stage. Although many vaccines at the development stage are able to achieve high clearance rates in mouse tumor models or even cases of total clearance, the clinical stage is still average. This necessitates the design of, for example, more intensive development of mRNA vaccines or combination vaccine-drug therapeutic regimens in terms of improving immune potency. As the most popular form of vaccine today, mRNA vaccines are highly immunogenic due to their mRNA status and the use of LNP as a natural adjuvant, making this form of vaccine highly effective. Currently, mRNA vaccines account for a very small proportion of HPV therapeutic vaccine development, and there is a trend for more consideration of this vaccine form in the future. In addition, the combination of vaccines and drugs is an important treatment option to enhance the effect, the most classic one being the combination with immune checkpoint inhibitors (Pardoll, 2012). For example, the drugs pembrolizumab and nivolumab, which are both anti-PD-1 antibodies, have shown some effectiveness in combination with HPV vaccines (Wendel Naumann and Leath, 2020). Currently, genetically engineered T-cell therapy is an emerging cancer treatment strategy, divided into CAR-T (Chimeric Antigen Receptor Engineered T cell) and TCR-T (T Cell Receptor Engineered T cell) therapies that have shown efficacy in hematologic cancers (Gao et al., 2019). In one study, they identified a high-purity TCR that targets HPV-16 E7 by recognizing the E7_11-19_ epitope complex. They therefore developed TCR-T therapy for the treatment of metastatic HPV-associated epithelial cancer, and tumor regression was observed in 6 of the 12 patients treated in the phase I clinical trial (Nagarsheth et al., 2021). This also provides an idea for the treatment strategy combined HPV vaccines and genetically engineered T-cell therapy. The use of adjuvants is also known to allow for a high therapeutic boost in vaccine efficacy, for example the use of Poly-ICLC adjuvant mentioned above allowed for 100% tumor regression in some experimental groups, in the future we need to strengthen the knowledge of Poly-ICLC and explore more efficient adjuvants. Second, to explore new therapeutic targets, most of the current vaccines focus mainly on E6 and E7 antigens, mainly because the continued increase in E6/E7 expression advances tumor progression. There are also a few vaccine developments focusing on E2 antigens for the treatment of precancerous lesions and symptoms such as genital warts. However, it has always been ineffective for cancer treatment, thus requiring the search for other new targets. E1 is required for viral replication and is the largest protein in HPV (E1 of HPV16 is 649aa), which most likely has a higher number of potential T-cell epitopes in the HLA allele compared to the smaller E6 and E7 proteins (154aa and 98aa for HPV16, respectively) (Boilesen et al., 2021). Moreover, additional studies have recently pointed to role in carcinogenesis of E1, which allows us to put it into the ranks of therapeutic vaccine design targets. It is similar to E2 for the development of vaccines for precancerous lesions, but what is most anticipated about it is the possibility of developing future therapeutic regimens against cancer. For E5, which is also a promising target for the development of therapeutic vaccines. Experiments have identified CD8 T cells in patients with HPV-positive head and neck cancer and identified several epitopes derived from HPV E5 protein, thus incorporating E5 as a vaccine antigen to trigger more tumor-reactive CD8 T cell responses (Eberhardt et al., 2021). For E4, little research has been done on them in the past.

In summary, future directions for HPV therapeutic vaccines include the development of new potent adjuvants, new antigen targets, and enrichment of preclinical models.

## 6 Conclusion

Diseases caused by HPV infection have always been the focus of human attention, and they are the necessary factors that cause cervical cancer (100%). Vaccines are currently playing a critical and effective role in the prevention and treatment of cervical cancer. Over the past 20 years, vaccine research has evolved rapidly, with the licensing of bivalent, quadrivalent and nonavalent vaccines effectively preventing 90% of HPV infections worldwide. However, research on new prophylactic vaccines has never stopped, as evidenced by the exploration of new expression systems, such as *E. coli*, to reduce the cost of vaccines, and the development of L2 vaccines with broad-spectrum protection for simplicity and efficiency. In the treatment of cervical cancer, surgery is currently the main treatment, and a therapeutic vaccine has not been licensed for marketing. Progress in the development of therapeutic vaccines is promising, with vaccines mostly designed on the basis of the tumor proteins E6/E7 to induce strong cellular immunity and hopefully eradicate HPV-related diseases and malignancies. Vaccines are generally effective in preclinical studies, but not in the clinical phase. The efficacy of the vaccine will also be improved in the future by exploring more *in situ* tumor models, combination drug therapy, and designing new antigenic targets (such as E1 and E5). We hope that therapeutic vaccines will be available soon.

## Author Contributions

YM, JM, YX, HZ, JS, JH and JC wrote the manuscript. YM, CQ reviewed and edited the manuscript before submission. JM prepared the figures and tables. All authors contributed to the article and approved the submitted version.

## Funding

This work was supported by National Key Research and Development Program (SQ2020YFF0401839), and National Natural Science Foundation of China (91959206).

## Conflict of Interest

Author JS was employed by the company Chongqing Precision Biotech Co., Ltd. Authors JM, HZ, YX, JC and JH were employed by the company Chongqing Institute of Precision Medicine and Biotechnology Co., Ltd.

The remaining authors declare that the research was conducted in the absence of any commercial or financial relationships that could be construed as a potential conflict of interest.

## Publisher’s Note

All claims expressed in this article are solely those of the authors and do not necessarily represent those of their affiliated organizations, or those of the publisher, the editors and the reviewers. Any product that may be evaluated in this article, or claim that may be made by its manufacturer, is not guaranteed or endorsed by the publisher.
